# Dengue fever presenting with acute cerebellitis: a case report

**DOI:** 10.1186/1756-0500-7-125

**Published:** 2014-03-05

**Authors:** Milinda Withana, Chaturaka Rodrigo, Thashi Chang, Panduka Karunanayake, Senaka Rajapakse

**Affiliations:** 1 University Medical Unit, National Hospital, Colombo, Sri Lanka; 2 Department of Clinical Medicine, Faculty of Medicine, University of Colombo, Colombo, Sri Lanka; 3 Department of Clinical Medicine, Faculty of Medicine, University of Colombo, 25, Kynsey Road, Colombo 08, Sri Lanka

## Abstract

**Background:**

The incidence of dengue fever is on the rise in tropical countries. In Sri Lanka, nearly 45,000 patients were reported in 2012. With the increasing numbers, rare manifestations of dengue are occasionally encountered. We report a patient who presented with bilateral cerebellar signs as the presenting feature of dengue.

**Case presentation:**

A 45-year-old previously healthy female from the suburbs of Colombo, Sri Lanka presented with an acute febrile illness associated with unsteadiness of gait. Clinical examination revealed a scanning dysarthria and marked horizontal nystagmus with bilateral dysmetria, dysdiadokokinesia and incordination more prominent on the right. Her gait was wide-based and ataxic with a tendency to fall to the right more than to the left. Dengue nonstructural protein antigen 1 test and IgM antibody testing both became positive indicating acute dengue infection. She recovered from the febrile episode within 9 days since the onset of fever but cerebellar symptoms outlasted the fever by one week. The magnetic resonance imaging of brain was normal and cerebellar signs resolved spontaneously by day 17 of the illness.

**Conclusions:**

Cerebellar syndrome in association with dengue fever has been reported in only four instances and our patient is the first reported case of dengue fever presenting with cerebellitis as the first manifestation of disease. This case report is intended to highlight the occurrence of acute cerebellitis as a presenting syndrome of the expanding list of unusual neurological manifestations of dengue infection.

## Background

Dengue fever is a common arboviral infection in the tropics; resulting in significant morbidity and, occasional mortality. Its incidence is on the rise in many tropical countries with periodic peaks of epidemic proportions reported following monsoon rains. In 2012, 44,456 cases of dengue fever were reported in Sri Lanka [1]. This is likely to be an underestimate given that serological tests of dengue fever were not readily available in state-owned hospitals during 2012 and patients with uncomplicated illness are often not hospitalized.

Classical dengue fever presents as a febrile illness with an uneventful recovery. A proportion of patients develop potentially life-threatening dengue haemorrhagic fever, which is associated with plasma leakage and shock [2]. Acute liver failure, acute kidney injury, and multi-organ failure are known complications [2]. However, many unusual manifestations have been reported with dengue, and there are many reports of neurological manifestations. These include aseptic meningitis, encephalitis, myelitis, intracranial haemorrhage and mono/polyneuropathies [3]. The pathophysiological basis of these neurological manifestations is not fully understood. Unusual manifestations are likely to be encountered more often in regions where the incidence of disease is high resulting in diagnostic confusion.

Acute cerebellitis is known to occur as an immune-mediated complication following varicella and coxsackie virus infections. Cerebellar involvement in dengue infection is not clearly defined. We report a patient who presented with a cerebellar syndrome as the initial manifestation of dengue fever adding to the expanding list of unusual manifestations of dengue infection.

## Case presentation

A 45-year-old previously healthy female from the suburbs of Colombo, Sri Lanka presented to a general medical unit of The National Hospital of Sri Lanka (NHSL) with an acute febrile illness associated with unsteadiness of gait. Three days prior to admission, the patient developed high fever with chills, arthralgia, myalgia and moderate headache worse in the morning. During the course of the same day, she found that she had difficulty in walking due to unsteadiness and intermittent dizziness. She did not have any other focal neurological signs or an apparent source of infection.

On admission, she looked ill and was febrile (101.5°F). Her pulse rate was 88 beats per minute; blood pressure was 110/70 with no postural hypotension; respiratory rate was 14/minute. Mild bilateral conjunctival injection was noted, although no haemorrhages were seen. There was no neck stiffness, skin rash, lymphadenopathy or arthritis. On neurological examination she was alert and oriented with a Glasgow coma scale (GCS) score of 15/15. She had a scanning dysarthria and marked horizontal nystagmus with bilateral dysmetria, dysdiadokokinesia and incordination which was more prominent on the right. Her gait was wide-based and ataxic with a tendency to fall to the right more than to the left. The rest of the neurological examination including tone, power, reflexes and sensation was normal.

Her investigation results on admission were as follows: leucocyte count 4450/mm^3^; neutrophils 3070/mm^3^, lymphocytes 850/mm^3^, monocytes 400/mm^3^; platelets 118,000/mm^3^; hemoglobin 11.7 g/dl, hematocrit 33.4%; erythrocyte sedimentation rate 8 mm/hour. Blood film done a day later showed leukopenia, lymphocytosis and thrombocytopenia suggestive of an acute viral infection. Renal function, electrolytes, blood glucose were normal. Liver transaminases showed a 3 fold rise above the upper limit of normal (alanine aminotransferase; 136 U/l, aspartate aminotransferase; 140 U/l). Non-structural protein 1 (NS1) test for dengue antigen was positive.

Her fever subsided by the fourth day of the illness, but the cerebellar symptoms and signs persisted. The platelet count continued to drop reaching a nadir of 36,000/mm^3^ by day seven of illness. However, no evidence of plasma leakage or shock occurred. She was managed with oral fluid replacement. On day nine of the illness, her constitutional symptoms resolved completely.

Dengue IgM and IgG antibodies by enzyme-linked immunosorbent assay (ELISA) were positive on the seventh day of the illness. Mycoplasma, herpes simplex virus, Epstein Barr virus, human immunodeficiency virus and cytomegalovirus antibodies were not detected. The Venereal Disease Research Laboratory (VDRL) test was negative. Anti-nuclear cytoplasmic antibody was negative. Magnetic resonance imaging (MRI) of the brain with gadolinium contrast (performed at day 7 of the illness) was normal. The patient refused consent for lumbar puncture.

She was discharged from hospital on day 9 when her platelet count returned to normal. Her cerebellar signs progressively improved had resolved completely by D17 when she was reviewed in the follow up clinic.

## Discussion

Neurological complications occur in 0.5-6.0% of patients with dengue infection [4]. Immune mediated mechanisms as well as direct tropic effects of the virus have been postulated to cause these neurological manifestations, and dengue antigen has been demonstrated in the brain in some patients with dengue encephalitis [5]. Post-infectious cerebellar syndrome has been described following several viral infections, but the association with dengue has been reported in only four instances [6,7]. In these reports, the onset of cerebellar symptoms varied between two days to two weeks after the onset of fever [6,8]. Our patient is the first reported case of dengue fever presenting with cerebellitis as the first manifestation of disease. Interestingly, the cerebellar syndrome outlasted the fever by almost two weeks. This report also highlights the self-limiting nature of this rare but severe neurological complication of dengue infection. It is noteworthy that in our patient, the MRI scan of the brain was normal. Of four patients previously reported in literature with dengue associated cerebellar features, the MRI showed hyper-intensity of cerebellum in T2 sections in two patients while it was normal in the other two [6,7]. One of the patients who had cerebellar hyperintensity on imaging had Epstein Barr virus co-infection [7]. MRI evidence of acute cerebellitis therefore may be transient and hence not seen in every patient depending on the timing of imaging.

Acute cerebellitis in relation to virus infection can be primary-infective or post-infective [9]. Acute primary infective cerebellitis mostly occurs secondary to infections such as varicella zoster virus, Epstein–Barr virus, measles, mumps, rubella, herpes simplex virus and coxsackie virus. Post-infective cerebellitis have been reported following infection with varicella zoster virus, coxsackie virus, Epstein–Barr virus and human immunodeficiency virus. Given the temporal profile of presentation, it is likely that our patient represents primary-infective cerebellitis following dengue infection.

## Conclusion

This case report is intended to highlight the occurrence of acute cerebellitis as a presenting syndrome of the expanding list of unusual neurological manifestations of dengue infection.

## Consent

Written informed consent was obtained from the patient for publication of this case report. A copy of the written consent is available for review by the Editor-in-Chief of this journal.

## Competing interests

The authors declare that they have no competing interests.

## Authors’ contributions

All authors were involved in the management of patient. MW wrote the first draft. CR, SR and TC revised it. All authors have read and approved the final manuscript.

## Authors’ information

MW (MBBS) is a registrar in Medicine attached to the University Medical Unit of National Hospital of Sri Lanka. CR (MBBS, MD) is a lecturer in Medicine, Department of Clinical Medicine, Faculty of Medicine, University of Colombo. TC (MBBS, MD, FRCP, DPhil) and PK (MBBS, MD, FRCP) are Senior Lecturers attached to the same unit. SR (MBBS, MD, FACP, FRCP, FRCPE, FCCP) is the Professor in Medicine, Faculty of Medicine, University of Colombo.

## References

[B1] Dengue cases by month 2012 [http://www.epid.gov.lk/web/index.php?option=com_casesanddeaths&]

[B2] Rajapakse S Rodrigo C Rajapakse A Treatment of dengue fever Infect Drug Resist 2012 5 103 112 22870039 10.2147/IDR.S22613 PMC3411372

[B3] Gulati S Maheshwari A Atypical manifestations of dengue Trop Med Int Health 2007 12 1087 1095 10.1111/j.1365-3156.2007.01891.x 17875019

[B4] Hendarto SK Hadinegoro SR Dengue encephalopathy Acta Paediatr Jpn 1992 34 350 357 10.1111/j.1442-200X.1992.tb00971.x 1509881

[B5] Lum LC Lam SK Choy YS George R Harun F Dengue encephalitis: a true entity? Am J Trop Med Hyg 1996 54 256 259 8600761 10.4269/ajtmh.1996.54.256

[B6] Weeratunga PN Caldera HP Gooneratne IK Gamage R Perera WS Ranasinghe GV Niraj M Spontaneously resolving cerebellar syndrome as a sequelae of dengue viral infection: a case series from Sri Lanka Pract Neurol 2013 [Epub ahead of print] 10.1136/practneurol-2013-000571 23840070

[B7] Karunarathne S Udayakumara Y Fernando H Epstein-Barr virus co-infection in a patient with dengue fever presenting with post-infectious cerebellitis: a case report J Med Case Rep 2012 6 43 10.1186/1752-1947-6-43 22289296 PMC3284420

[B8] Verma R Sharma P Garg RK Atam V Singh MK Mehrotra HS Neurological complications of dengue fever: experience from a tertiary center of north India Ann Indian Acad Neurol 2011 14 272 278 10.4103/0972-2327.91946 22346016 PMC3271466

[B9] Sawaishi Y Takada G Acute cerebellitis Cerebellum 2002 1 223 228 10.1080/14734220260418457 12879984

